# Stem Cells and Extrusion 3D Printing for Hyaline Cartilage Engineering

**DOI:** 10.3390/cells10010002

**Published:** 2020-12-22

**Authors:** Océane Messaoudi, Christel Henrionnet, Kevin Bourge, Damien Loeuille, Pierre Gillet, Astrid Pinzano

**Affiliations:** 1 UMR 7365 CNRS-UL, IMoPA (Ingénierie Moléculaire et Physiopathologie Articulaire), Biopôle de l’Université de Lorraine, Campus Brabois-Santé, 9, Avenue de la Forêt de Haye, BP20199, 54505 Vandœuvre-Lès-Nancy, France; oceane.mess.96@gmail.com (O.M.); christel.henrionnet@univ-lorraine.fr (C.H.); kevin.bourge@univ-lorraine.fr (K.B.); d.loeuille@chru-nancy.fr (D.L.); pierre.gillet@univ-lorraine.fr (P.G.); 2 Service de Rhumatologie, CHRU de Nancy, Hôpitaux de Brabois, Bâtiment des Spécialités Médicales, 5 rue du Morvan, F54511 Vandœuvre-Lès-Nancy, France; 3 Laboratoire de Pharmacologie, Toxicologie et Pharmacovigilance, Bâtiment de Biologie Médicale et de Biopathologie, CHRU de Nancy-Brabois, 5 Rue du Morvan, F54511 Vandœuvre-Lès-Nancy, France; 4 Contrat d’Interface, Service de Rhumatologie, Hôpital de Brabois, Bâtiment Spécialités Médicales, F54511 Vandœuvre Lès Nancy, France

**Keywords:** stem cells, 3D printing, cartilage engineering, bio-ink

## Abstract

Hyaline cartilage is deficient in self-healing properties. The early treatment of focal cartilage lesions is a public health challenge to prevent long-term degradation and the occurrence of osteoarthritis. Cartilage tissue engineering represents a promising alternative to the current insufficient surgical solutions. 3D printing is a thriving technology and offers new possibilities for personalized regenerative medicine. Extrusion-based processes permit the deposition of cell-seeded bioinks, in a layer-by-layer manner, allowing mimicry of the native zonal organization of hyaline cartilage. Mesenchymal stem cells (MSCs) are a promising cell source for cartilage tissue engineering. Originally isolated from bone marrow, they can now be derived from many different cell sources (e.g., synovium, dental pulp, Wharton’s jelly). Their proliferation and differentiation potential are well characterized, and they possess good chondrogenic potential, making them appropriate candidates for cartilage reconstruction. This review summarizes the different sources, origins, and densities of MSCs used in extrusion-based bioprinting (EBB) processes, as alternatives to chondrocytes. The different bioink constituents and their advantages for producing substitutes mimicking healthy hyaline cartilage is also discussed.

## 1. Introduction

Articular cartilage is a specialized tissue that lines the ends of the epiphyses and allows joint movement. It is a layered tissue consisting of 4 zones—the superficial, transitional, deep, and calcified areas separated from the underlying bone. Differences in cell morphology, the constitution of the extracellular matrix (ECM), and the collagen fibers’ orientation in each layer are responsible for the overall structure’s physical and biomechanical properties. The primary cartilage cell is the chondrocyte, whose prominent role is to maintain the ECM’s integrity. The physiology, morphology, and metabolism of the chondrocyte also vary from one area to another. Unfortunately, cartilage has limited self-repair capabilities due to its avascular nature. Focal or diffuse cartilage damages can lead to pain, joint dysfunction, or even secondary osteoarthritis. Apart from arthroplasty, the repair or replacement of hyaline cartilage is a significant challenge in orthopedic surgery. Current orthopedical methods such as microfracture, autologous chondrocyte implantation, or mosaicplasty might offer short-term solutions. Still, none of them provide lasting repair, as the quality of the scarred neocartilage remains poor. As such, tissue engineering presents itself as a promising alternative for the restoration of cartilage lesions.

Joint engineering is an interdisciplinary field that aims to recreate a neo-tissue whose physical and biochemical property are close to those of the native tissue. It combines cells, biomaterials, and environmental factors. It represents a potential tool for cartilage regeneration. The main criteria to be taken into account are:

- homogeneous distribution of cells into the biomaterial,
- adapted porosity for homing, nutrient diffusion,
- cell differentiation optimized towards chondrocytes-like cells,
- preservation of the chondrocyte phenotype in situ,
- synthesis of a peri-cellular chondral-like ECM,
- persistent cell viability despite progressive biodegradation of the bioprinted biomaterial,
- biomechanical properties progressively close to those of the native cartilage
- bio-integration of the implants into the joint.

It is worth noting that conventional cartilage engineering methods have minimal control over the shape, size, and organization of engineered products.

In contrast, the development of 3D bio-imaging technologies represents a recent revolution in personalized regenerative medicine. This technique makes it possible to obtain a well-defined, sometimes complex structure of a custom size, using a layer-by-layer bio-manufacturing strategy, guided by computer-aided design. Different 3D bioprinting processes are available, depending on the mechanical and biochemical properties of the native tissues. The three primary techniques currently developed are laser-assisted printing, inkjet, and bio-extrusion. The restoration of a layered structure such as a native hyaline cartilage is a complicated technological lock. The bio-extrusion process presents itself as the best alternative to recreate layered structures, such as skin and cartilage tissue. One of the advantages is the direct encapsulation of living cells in the bio-ink, during the printing process, which allows the production of customized composite biomaterials. In this review, after a brief synopsis of normal and pathological cartilage, as well as the leading 3D printing methods, we focused on the interest of bioextrusion in cartilage engineering, according to the biomaterial composition of the bioinks, and the nature of the cell contingent, mainly mesenchymal stromal stem cells (MSCs), which are pluripotent, depending on the environment used.

**Figure 1:**
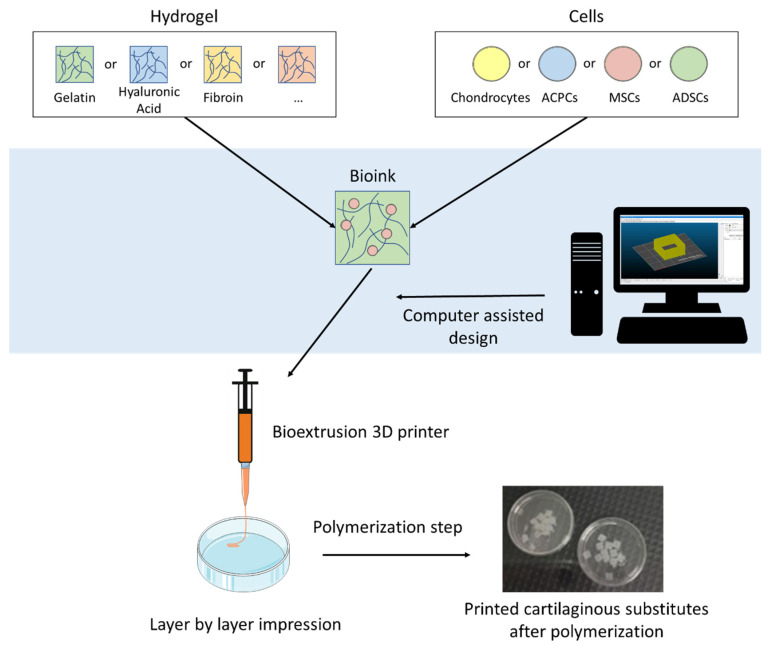
Schematic illustration of the extrusion-based 3D bioprinting process for articular tissue engineering using various hydrogels and cell types.

To this day, many exhaustive review already exist concerning the use of 3D printing for the reconstruction of various tissues [1,2], or specifically on 3D printing for cartilage regeneration [3,4]. The originality of our work is the particular focus on extrusion bioprinting of cellularized hydrogels for articular cartilage tissue engineering (Figure 1).

## 2. Methodology

To perform this review, we searched for articles published up to 1 December 2020, in PubMed, with no start date restriction, with the keywords ‘3D printing cartilage’. About 442 references were found. Second, a manual search of reference lists from selected articles was conducted, with the keywords ‘3D printed AND cartilage’, ‘extrusion AND cartilage’, ‘biofabrication AND cartilage’, ‘3D printing AND chondrogenesis’ ‘extrusion AND chondrogenesis’ and ‘biofabrication AND chondrogenesis’. We only selected the references that specifically uses a bioextrusion process of a cellularized hydrogel. The studies using a PCL scaffold for support, post-printing molding, or aiming to recreate tissues other than articular cartilage were eliminated. Only 25 were selected based on the selected criteria. We apologize for those excluded from those criteria, and therefore not cited in this review.

## 3. Articular Cartilage Lesions and Their Surgical Treatment

### 3.1. Osteoarthritis

Osteoarthritis (OA) is the most common joint affliction, and its frequency and socioeconomic impacts make it a public health challenge around the world, particularly in the context of overall population aging [5]. Its symptoms are pain, swelling, occasionally inflammation, and articular rigidity [6]. It is characterized by progressive degeneration of cartilage that can lead to subchondral bone damage. Cartilage loss causes bone remodeling, which is associated with acute pain [7]. There are four stages of pathological attack, depending on the extent of the remodeling [8,9]. OA management encompasses the prevention and treatment of pain, and includes palliative measures such as anti-inflammatory drugs and analgesics [10,11]. Pharmacological treatments are usually paired with physical therapy and weight control, to maintain or improve joint function [6,12,13]. When those measures fail to improve the patient’s lifestyle, the last resort is surgery to substitute the damaged joint with a synthetic prosthetic [10]. Early OA is characterized by a lack of existing lesions. The exact causes of its appearance are still unknown [14]. Some already identified factors are age, sex, weight, and metabolic dysfunction [11,15,16]. Secondary arthrosis follows repetitive or abnormal loading; traumas can damage articular chondral surfaces [17]. These traumas can lead to mechanical dysfunction, swelling, or pain. The depth of their focal lesion classifies the different articulation injuries—(1) chondral lesion leading to mechanical malfunction and (2) osteochondral lesion with damaged cartilage, and subchondral bone causing articular disruption [18].

In chondral lesions, only the articular cartilage is injured. Chondrocytes near the lesion react by increasing the synthesis of extracellular matrix proteins, but due to their low proliferation rate, the cells are unable to restore cartilage integrity [19]. The increased synthesis is quickly stopped, leaving the articular surface with a chondral defect that can degenerate [20]. When the damage reaches the subchondral bone, undifferentiated cells from the bone marrow can infiltrate the injured zone to start the healing process. Mesenchymal stem cells (MSCs) differentiate into chondrocyte-like cells and initiate extracellular matrix synthesis (ECM), but the organization and biomechanical properties of this newly synthesized matrix differ from those of hyaline cartilage [20]. This fibrous repair tissue is unstable and leads to long-term degradation of the articular surface and function. Cartilaginous defects tend to progress over time and might lead to OA [21,22]. One of the significant challenges of cartilage regenerative medicine is treating these traumatic cartilage lesions early, to prevent long-term degradation and secondary OA.

### 3.2. Actual Management and Its Limitations

Numerous surgical techniques were developed to address focal cartilage defects [23]. Here, we present some of the most commonly used surgical procedures and their processes. Abrasion is a technique developed by Johnson in 1980 [24]; it is based on superficial debridement of the exposed bone to expose the vascularity underneath and obtain a viable bone surface to permit fibrin clot formation and attachment. The newly formed tissue on the exposed bone surface is a fibrocartilage-type tissue resulting from blood clot differentiation [25]. Microfracture and similar methods aim to stimulate the natural healing properties of the body. Microfracture involves the perforation of the subchondral bone, to allow MSCs and growth factors to escape the bone and fill the defect with newly synthesized tissue. Microfracture can only be applied to treat full-thickness defects with healthy subchondral bone. Furthermore, the repair is made of fibrocartilaginous tissue and is not stable in the long-term, generally leading to joint surface degradation [26]. Full-thickness osteochondral grafts are usually allografts. In this case, a unique cylindrical sample is harvested from a tissue donor and reimplanted in the defect to fill the lesion. This method allows for partial reparation of the surface through the formation of fibrocartilage between the native tissue and graft [27].

In mosaicplasty, multiple cylindrical cartilage grafts are harvested from a healthy, nonbearing zone of the patient’s joint [28], and then reimplanted to fill the defect. This surgical procedure aims to permit repair of the articular surface, by producing neocartilage in the gaps separating the edges of the lesion and cartilage shreds. It causes donor-site morbidity but provides good long-term stability [29]. Brittberg’s technique, or autologous chondrocyte implantation (ACI), was first performed in 1987 [30]. It consists of multiplying the patient’s chondrocytes in vitro and then reinjecting them into the injured area with support, allowing them to fill the cartilaginous defect. These different techniques generally result in insufficient quality repair tissue, with low type II collagen content. This fibrocartilaginous tissue does not possess the phenotype of native hyaline cartilage [31] and might not support the necessary constraints and biomechanical loads. Hence, finding alternatives to those surgical procedures is a public health issue challenge. Tissue engineering (TE) approaches offer the potential to recreate hyaline-like cartilage in vitro, making them a promising tool for cartilage rehabilitation.

### 3.3. Healthy Cartilage Structure and Composition

Articular cartilage is a living, specialized connective tissue found in diarthrodial joints such as knee or hip joints. Its primary role is to provide a smooth and lubricated surface to permit load transmission during movement with a low friction coefficient [32]. Cartilaginous tissues can support movement and resist shear stress and deformation. These tissues need to store energy to prevent lasting compression [33,34]. Cartilage is a nonvascular and noninnervated tissue that possesses limited self-regeneration properties. It comprises a single cell type, chondrocytes, and a dense extracellular matrix. Thus, the sole resident cells of the cartilage are chondrocytes. Chondrocytes represent only 10% of the articular cartilage tissue volume [35]. The chondrocytes are spread across a dense matrix and have no cell-to-cell contact. They are responsible for the synthesis and degradation of the ECM component and maintain the homeostasis of the tissue; they secrete integrins as mediators to control cell differentiation, proliferation, and survival and matrix remodeling [36]. Chondrocytes specifically synthesize proteoglycans (PGs), collagens, and other noncollagenous proteins [32,37]. Chondrocytes are isolated in hypoxic niches, making hypoxia-inducible factor 1-alpha a key regulator of differentiation and homeostasis [38].

The hyaline ECM comprises water, PGs, and collagens, particularly type II collagen. To a lesser extent, noncollagenous proteins and glycoproteins are present in the ECM [39]. The cartilaginous matrix is highly hydrated (65 to 80% of the total weight) with a specific repartition of the water between the intra- and extrafibrillar compartments [33]. Less than one-third of the water content is linked with collagenous fibrils, the rest is located in the free space of the ECM [40]. PGs are highly glycosylated proteins, composed of a core protein and glycosaminoglycan chains, such as chondroitin sulfate (CS) or keratan sulfate [33,41]. The most common PG in cartilage is aggrecan, which plays a role in load-bearing. PG binds hyaluronan, forming a complex that retains a high amount of water in the ECM [37,42]. Collagen is a fibrillar protein; its primary role is to form a complex and organized network supporting the matrix structure [43,44]. Type II collagen is one of the main components of the ECM. It is distributed in a gradient with a higher density in the superficial zone of the hyaline cartilage and the lowest density in the deep zone. Type II collagen interacts with other collagen types, such as IX, XI, and III, for structural purposes [45].

Hyaline cartilage possesses a unique zonal organization in four differentiated layers [32]. The superficial zone is the thinnest layer, containing collagen fibers (primarily type II and IX) oriented parallel to the articular surface. This layer is responsible for most of the resistance properties of the articular surface [39]. In the transitional zone, collagen fibrils are thicker and less organized, with more PGs [35]. The deep zone is characterized by the highest density of PGs and the lowest water content; the collagen structures perpendicular to the tidemark separate the deep zone from the subchondral bone [46]. The chondrocyte density, morphology, and gene expression also vary depending on the depth within the hyaline cartilage [47,48,49]. These differences are also associated with the different biomechanical stresses exerted on the different layers [43,50]. This particular zonal organization and cellular distribution are a crucial component of cartilage tissue repair. The reconstruction of cartilaginous tissue with good repair, integration, and biomechanical properties is the main challenge of cartilage tissue engineering.

### 3.4. Tissue Engineering for Cartilage Repair

Cartilage tissue engineering is currently considered a promising alternative to classic treatment strategies [51,52,53]. It aims to recreate cartilaginous substitutes with properties similar to those of natural cartilage. The three main axes to consider for cartilage TE are biomaterials, cells, and the environment [54]. The biomaterial needs to have optimal porosity, reticulation, biointegration, cell-seeded scaffolds, cytocompatibility, and good cell adhesion properties [55,56]. Different cell types were investigated for cartilage tissue engineering, the most common being chondrocytes and MSCs. The last important factor is the environment. Chondrogenic matrix synthesis is driven by growth factors [57,58,59,60,61], oxygen levels [62,63,64,65,66], maturation time [67], and mechanical stimuli, including dynamic compression and shear stress, to mimic the natural diarthrodial environment [68,69,70,71,72,73]. Classical TE usually produces homogenous constructs. A new field of tissue engineering development is the field of 3D printing. It offers new potential to produce stratified products as well as innovations in personalized regenerative medicine.

## 4. 3D Printing for Cartilage Tissue Engineering

### 4.1. History of 3D Printing 

Three-dimensional (3D) printing (or additive manufacturing) was invented in 1983 by Chuck Hull. Initially, the printing process was based on the stereolithography method. A computer-aided design (CAD) is created and sent to a 3D printer. Sequential coats of material are solidified in a layer-by-layer manner, until the full product is produced [74]. Numerous 3D printing processes were developed, based either on the solidification of materials via an energy source or by the deposition of a liquid material that is polymerized postprinting. First used exclusively in industry, 3D printing reached the medical field in the 2000s to produce synthetic surgical models. Subsequently, the concept of bioprinting emerged, raising the possibility of printing biological tissues and organs [75]. A decade later, the first printing process with live cell structures was successfully executed [76]. The concept of bioprinting is a promising perspective for modern tissue engineering. It has only begun to influence medicine and surgery and revolutionize health care [77].

### 4.2. 3D Printing Processes for Cartilage Reconstruction

Significant advances were made in the field of cartilage and bone tissue engineering over the past two decades [3]. 3D printing is a revolutionary process for the field of personalized medicine. It allows for the layer-by-layer deposition of a biomaterial as specified by a CAD, making it possible to adapt the constructs to specific lesions, unlike other classical TE methods [78,79]. Two main printing classes can be distinguished—acellular processes and bioprinting. In this review concerning articular cartilage engineering, we focus on bioprinting strategies.

**Figure 2:**
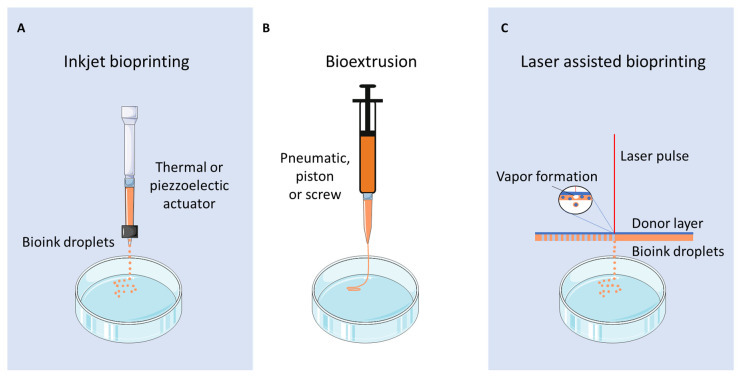
Different bioprinting strategies are available for cartilage tissue engineering. (**A**) Inkjet bioprinting based on the deposition of droplets formed by a thermal or piezoelectric actuator on a support. (**B**) Bioextrusion (or microextrusion) based on the extrusion of a continuous bioink filament through a printing needle driven by a screw, pneumatic or a piston. (**C**) Laser-assisted bioprinting uses a laser-based energy source to produce droplets via the production of a vapor bubble in the substrate.

Different bioprinting strategies are available for cartilage tissue engineering—inkjet printing, laser-assisted printing, and bioextrusion (Figure 2) [80]. Inkjet bioprinting is based on the deposition of droplets directly onto a support by thermal or piezoelectric methods [81]. Laser-assisted bioprinting consists of the deposition of droplets from a specific material onto a receiving substrate, under the influence of a laser-based energy source [82]. Bioextrusion (or microextrusion) is a process based on the direct deposition of a bioink onto a support via a printing needle (screw-based, pneumatic, or piston-driven), following a CAD [83]. Bioextrusion is used in association with bioinks composed of natural (alginate, gelatin, chitosan, hyaluronan) or synthetic (PCL, PGA, PEG) polymers and embedded cells.

The bioextrusion process allows the printing of large and bulky substitutes with high cell density, making it an excellent candidate to reproduce full-thickness cartilage tissue [84,85]. It allows the deposition of different biomaterials and cell types throughout the different printed layers of bioink to better mimic the natural osteochondral organization, and more precisely, the four different hyaline cartilage layers [86]. This innovative process could allow for personalized constructs with the zonal organization of native cartilage directly adaptable to the patient’s lesion sites.

## 5. Bioextrusion Processes for Cartilage Tissue Engineering

### 5.1. Bioinks for Extrusion-Based Bioprinting

Biomaterials used for classic tissue engineering need to have specific characteristics. The main factors are biocompatibility, biodegradability, and porosity [87]. The biomaterial needs to have biomechanical properties compatible with those of the native tissue it aims to recreate. It also needs to be loose enough to permit ECM development but stable enough to maintain a three-dimensional environment for the cells [55]. The biomaterial needs to be adapted to the target tissue, the cell type, and mechanical constraints. Specifically in cartilage TE, biomaterials play different roles depending on the embedded cell types. The environments required to maintain chondrocyte differentiation and permit MSC differentiation induction are different [88,89,90]. The integration of the engineered substitute with the surrounding healthy tissue needs to be assessed.

The two main classes of biomaterials used for tissue engineering are synthetic and natural polymers. Synthetic polymers are human-made materials that are already widely used in cartilage tissue engineering for their well-characterized and stable chemical properties. Polymers such as poly(ethylene)-glycol (PEG), polycaprolactone (PCL), or polyglycolic acid (PGA) can be combined or coated with hydrogels or natural polymers to enhance their biocompatibility [91,92,93,94]. Another class of material developing is nanomaterials, such as carbon nanotubes (CNTs) for their physico-chemical properties [95]. Natural polymers are also considered promising for TE, with alginate, gelatin, and agarose being widely studied for their properties [96]. The different biomaterial concentrations can be tuned to optimize the final construct’s biological and mechanical properties [97,98]. They can be combined to form a complex bioink, taking advantage of their different biomaterial characteristics. To improve the stability of some natural polymers, such as gelatin or hyaluronic acid (HA), modifications such as methacrylation are often used [99,100]. Hydrogels are natural polymers widely used for their excellent biocompatibility and ECM mimetism [101]. Hydrogels are usually polysaccharides (e.g., alginate, HA) [102,103] or protein-based (collagen, fibrin) [89,103].

Hydrogels can also be based on a decellularized extra-cellular matrix (dECM). First used in biological sheets or coating for bioengineered scaffold, dECM can now also be used for cartilage 3D-printing [104]. The aim while producing dECM is to eliminate the cellular component, while maintaining the structure and composition of the native ECM [105]. It can easily be made into a soft gel, making it a promising feature for bioextrusion. The main advantage of using dECM as a biomaterial is the mimicking of the structure and biological cues of the native tissue that allows for the induction of growth and differentiation of the cellular contingent. For cartilage TE, dECM is already used as a bioink to produce 3D printed cartilaginous substitutes [106,107,108].

One crucial characteristic for any material used in three-dimensional bioprinting is printability. Bioinks designed for EBB processes are based on biocompatible and bioprintable hydrogels. The advantages of the different biomaterials used in EBB processes are presented in Table 1. Critical criteria include viscosity and viscoelasticity of the bioink to achieve the optimal printing process [109,110]. The stability and mechanical properties of printed gels are also essential considerations for the final construct [111]. To ensure three-dimensional stability, hydrogel-based bioinks can be solidified by temperature change, photocrosslinking, or chemical crosslinking [112,113,114]. Another method consists of printing a heterogeneous scaffold composed of a structural PCL scaffold and a cytocompatible hydrogel containing the cells [115,116].

The advantage of bioextrusion processes is the ability to print bioinks and cells to simultaneously produce functionalized substitutes. The biocompatibility of the bioink is often assessed by evaluating the viability of the printed cells [117,118,119]. In comparing different cell-seeded bioinks and printing parameters, the yield stress, shear stress, and viscosity were highlighted as crucial printing factors [120]. Shear stress can be impacted by different printing parameters, such as needle geometric shape and diameter [117]. These parameters need to be finely tuned to promote cell viability and differentiation potential in the final constructs. The biomaterial used to produce cellularized hydrogels also needs to be adapted to the construct’s cellular content.

### 5.2. Mesenchymal Stem Cells as an Alternative to Native Chondrocytes

As the sole resident of the cartilage, chondrocytes seem to be the most suitable cell type for cartilage tissue engineering. However, apart from the fact that they are hardly available within the joint, their amplification in a monolayer generates cell dedifferentiation from the first passages, with a significant decrease in type 2 collagen synthesis [121,122]. Of all adult stem cells present in the body, mesenchymal stem cells currently represent a promising candidate substitute for chondrocytes in cartilage tissue engineering. Many different factors characterize human MSCs, including their adhesion to plastic supports; their expression of stemness markers, such as CD105, CD73, CD29, and CD90; and their lack of expression of hematopoietic surface proteins CD45 or CD34 [123,124,125,126]. MSCs are self-renewable and multipotent-capable of differentiating into multiple cell lineages [127,128]. The signaling pathways that affect the differentiation of MSCs are well characterized [129]. Their multipotent potential can be influenced by their origin, depending on the cell source. Differences in chondrogenic and osteogenic properties were already highlighted [130,131,132]. First identified in 1970 by Friedenstein, MSCs were initially isolated from bone marrow [133], but further investigation showed that they could be easily isolated from other source tissues, the most common being adipose tissue [134], synovial membrane, synovial fluid [135,136], dental pulp [137], Wharton’s jelly, and umbilical cord blood [138].

Bone marrow-derived MSCs (BM-MSCs) are now well characterized because they are the most commonly used cell type in tissue engineering. They possess good proliferation properties but can also be induced to differentiate into various cell types, including osteocytes, adipocytes, chondrocytes, and neural or muscular cells [139,140]. To be induced in the chondrogenic lineage, they require a combination of differentiation factors (mainly growth factors such as TGF-β1 and TGF-β3) and a 3D environment to promote and stabilize the chondrogenic phenotype [57]. Adipose-derived stem cells (ADSCs) are widely used in cartilage tissue engineering for their chondrogenic properties [141,142,143]. Their main advantage over other MSCs is that they are easily accessible via minimally invasive procedures. Their differentiation capacity differs from that of BM-MSCs, which have better osteogenic properties, while ADSCs synthesize more collagen. They can be isolated from different fat tissues. Indeed, one of the sites most commonly used in cartilage TE is the infrapatellar fat pad, already in the knee joint [144,145].

MSCs are also present in the synovial membrane. Their multipotency was investigated to prove that they can differentiate into chondrogenic, osteogenic, adipogenic, and sometimes myogenic pathways [135]. They have healing potential for articular tears [146], making them good candidates for cartilage tissue engineering. Similar MSCs can be isolated from synovial fluid [59], presenting an MSC phenotype and surface markers. They possess the same multilineage potential as synovial membrane-derived cells. Synovial fluid mesenchymal stem cells show the highest chondrogenic potential among osteoarticular cell types [131]. MSCs isolated from dental pulp possess different differentiation properties. They primarily differentiate into the odontoblast pathway but can also be induced to become adipocytes, osteoblasts, chondrocytes, and neural cells [147,148]. Already used in cartilage TE, dental pulp MSCs exhibited potential for hyaline-like cartilage formation with the synthesis of ECM components, such as aggrecan or collagen [149,150,151]. Umbilical cord blood and Wharton’s jelly also contain mesenchymal-like cells expressing MSC markers and lacking hematopoietic markers [138,152]. Their multipotent potential for cartilage TE was already studied, and they showed good hyaline-like cartilage neosynthesis under different conditions with lower type X collagen synthesis than BM-MSCs [62,132,153].

As not all cell sources were used in EBB processes for hyaline cartilage regeneration, we focus only on the extruded cellularized constructs and their cell contingents.

### 5.3. Cell Types Used in Extrusion-Based Bioprinting

#### 5.3.1. Cartilage-Derived Cells

Chondrocytes are intensively investigated for cartilage regeneration. The EBB process allows for the direct bioprinting of chondrocytes embedded in bioinks to print cellularized constructs. They are usually associated with natural polymers. Some studies use alginate [117], HA [118], gelatin [154] or dECM-based bioinks [108]. Chondrocytes can be used to assess the biocompatibility and printability properties of different polymers and test different concentrations of polymers [120] or different biomaterial blends [155]. To further recreate the cartilage’s zonal organization, EBB systems were used to create layer-by-layer substitutes. The gradient can be tuned by modifying the cell density within the gridded construct [156]. Adding constituents to the deepest layers of the printed substitutes, such as calcium, can improve a calcified zone [157]. Once printed and matured, cartilaginous constructs can be used to assess chondrogenesis inside the biomaterial, by measuring gene expression and matrix synthesis [154]. Mechanical properties such as compressive stress are also key factors that need to be assessed to recreate native cartilage [158].

An alternative to mature chondrocytes for tissue engineering is a subpopulation of chondrocytes, articular chondroprogenitor cells (ACPCs) found in the surface zone of mature cartilage [159]. ACPCs maintain good chondrogenic potential after extending the monolayer culture, unlike mature chondrocytes [160]. ACPCs were already used in the EBB process developed with a gelatin-based bioink playing two key roles—the ink provides a scaffold to encapsulate the cells and acts as glue so that the extruded material directly adheres to the defect surface in situ [161]. ACPCs must be more deeply investigated to evaluate the chondrogenic potential of those cells. As seen previously, autologous chondrocytes are very limited in number, and while undergoing expansion in vitro, they might lose their phenotype, morphology, and expression of specific markers. Therefore, the limitations encountered in chondrocyte-based therapies instigated alternative cell searches as tools in cartilage regeneration.

#### 5.3.2. Mature MSCs

MSCs represent a promising alternative for cartilage tissue engineering due to their many advantages, as discussed previously. The most common MSCs used are BM-MSCs. By using extrusion-based 3D printing, BM-MSCs can be directly seeded into the biomaterial and extruded into a compact three-dimensional construct. Due to their potential, BM-MSCs could engineer different layers of native cartilage in vitro. MSCs are usually embedded in a hydrogel to reproduce the hyaline-like cartilaginous matrix, due to their excellent hydration properties, such as alginate [162] GelMA [163] or dECM-based bioinks [106]. An important aspect of tissue mimetism is the fiber organization within the different layers; the bioextrusion process can print layers with different alignments, making it possible to reproduce the collagen fibers’ natural organization within the cartilaginous ECM [103]. The addition of compounds that lead to differentiation was investigated at length. CS can induce cartilaginous matrix production, especially type II collagen, while the presence of HA in the hydrogel enhances cell viability and chondrogenesis [164]. HA also favors the hypertrophic differentiation of MSCs [165]. To further reproduce the calcified layer, calcium can be added to the bioink to increase the expression of hypertrophic cartilage markers [166]. To fine-tune the chondrogenesis of the embedded MSCs in 3D-printed constructs, the use of growth factors, especially TGF-β family members, is essential [60].

ADSCs can also be used in EBB processes to reproduce cartilaginous tissue through additive manufacturing. A specific device, the BioPen, was developed to directly print substitutes seeded with ADSCs [167]. Different studies assessed the process [168] and its potential for cartilage reparation therapy in vitro [143]. The next step was to directly print into a full-thickness defect with the handheld device, to assess in vivo printing in a large animal model, highlighting the ability of the ADSC combined with a bioextruded hydrogel, to promote the reparation of the cartilage by enhancing a more hyaline-like cartilaginous reparation [142]. Other MSC sources (synovium, Wharton’s jelly) were already used in classical cartilage tissue engineering, but their chondrogenic potential in an extrusion-based 3D printing process is yet to be assessed [146].

#### 5.3.3. Coculture of MSCs and Chondrocytes

The chondrocyte phenotypes vary from one zonal area to the next in joint cartilage [169]. To recreate this complex structural cellular organization, recent findings highlighted the fact that coculturing MSCs and chondrocytes in the same constructs could favor the induction of chondrogenesis, especially for BM-MSCs [170]. Studies showed that when combining a nanocellulose-based biomaterial with a coculture of BM-MSCs and chondrocytes at a ratio of 8:2, the presence of MSCs enhances the proliferation of chondrocytes in vivo [171]. Using the same parameters, it was also proven that MSCs could improve cartilaginous ECM synthesis in vivo, especially type II collagen synthesis [172]. To mimic the zonal organization of native cartilage, specific cell contingents can be used to reproduce different layers. Alginate and gelatin-based bioinks were used to produce a composite construct. The top layer comprises BM-MSCs and chondrocytes cocultured (7.5:2.5) with CS and is designed to reproduce hyaline-like cartilage. The bottom layer contains only MSCs embedded in a bioink to which HA was added to reproduce the calcified zone. These constructs demonstrate the potential for zone-specific cartilage tissue engineering [173]. Many factors in addition to cell origin need to be assessed to optimize neocartilage production in the constructs.

### 5.4. Cell Density for Cartilage Tissue 3D Printing

The cell seeding density to use during 3D bioprinting remains an open question. Due to chondrocytes’ low proliferation rate, a higher cell density generally yields a better engineered cartilage tissue [174]. The cell density used for the extruded products generally ranges from 5 to 20 × 10^6^ cells/mL. Very few studies aimed to compare those densities to assess induced chondrogenesis [156]. In most cases, researchers work with a standard density optimized within that range [108,117,118,119,120,155,157]. In one study working with chondroprogenitors, a high density of 20 × 10^6^ was also selected [161]. Very few studies used low cell densities, but they achieved good chondrogenic results [154,158]. For BM-MSCs, the range of densities is comparable to that of chondrocytes, starting from 4 × 10^6^ cells/mL [175] and increasing to as high as 20 × 10^6^ cells/mL [163]; most of the studies were between those two values, thus achieving the best chondrogenic induction possible [106,162,165,166]. Recently, some research aimed to compare two very low cell densities, 1 and 2 × 10^6^ cells/mL, to assess better options for obtaining good chondrogenesis, and they showed that the lowest density seemed to be optimal [60]. This could lead to a new approach in cartilage tissue engineering by better mimicking the chondrocytes’ natural repartition in cartilage.

ADSCs delivered with BioPen technology are seeded at a slightly lower range of densities than the other cell types, between 2 and 10 × 10^6^ cells/mL [142,143,168]. These conditions seemed optimal to obtain good chondrogenic properties of the final construct in vitro and in vivo. In cocultured substitutes, although the cell density used is crucial. The most important cell number is usually 10 × 10^6^ cells/mL. Another important aspect is the ratio between the two cell types and the use of a higher density of stem cells than chondrocytes in all printed substitutes. The ratio most often used is 8:2 [171,172]. The ratio between MSCs and chondrocytes can vary between the layers, depending on the desired characteristics [173]. The chondrogenic differentiation of the embedded cells is impacted by their origin, their density, the nature of the biomaterial they are seeded in and environmental factors.

### 5.5. Cell Viability

The cells’ ability to proliferate and differentiate inside the 3D-printed cartilaginous construct is directly correlated with cell viability. The cell viability of the embedded cells depends on the cell type and density used for 3D printing of cartilaginous constructs, as presented in Table 2. Printing parameters such as shear stress and pressure applied to the cells during the extrusion process can affect cell viability, proliferation, and chondrogenic properties. To reduce shear stress during extrusion, nozzle diameter and geometry are vital factors [117]. Other printing parameters, such as cross-linking (UV light, ionic, or enzymatic gelation), also need to be tuned to permit maximum cell survival [60,103,119,165,166]. To assess the long-term effects of the overall printing process, many researchers evaluated viability at different times postprinting [162,165,166]. The biomaterials and additives in the bioink can also affect cell viability, exerting a cytotoxic effect on the embedded cells. Previous studies highlighted that adding components to the bioink, such as nanocellulose or calcium derivative, can reduce the cell viability [117,119,157,173]. This decrease can be explained either by a slightly toxic effect of the added molecule [157,173] or by a too highly concentrated hydrogel [118]. In contrast, other biomaterials, such as gelatin, silk, or collagen, can improve cell nesting and survival [154,155]. Other studies used cell aggregates or spheroids to seed into the bioink instead of single cells [103,175]. A comparison of embedded spheroids to isolated cells in bioextruded substitutes demonstrated a better survival of cells inside spheroids [175]. Globally, the bioextrusion process using biocompatible biomaterials and optimized printing parameters maintains good viability, generally as high as >90% viability, after a sufficient maturation time [108,118,142,156,163,168,173].

### 5.6. Environmental Factors

First, oxygenation plays a crucial role in the chondrogenic pathway. The articular cartilage is avascular; the chondrocytes are therefore exposed to low oxygen contents, which vary from 5% at the level of the superficial zone to 2% in the deep zone [176]. The surrounding medium’s oxygen content impacts the proliferation, maintenance of the chondrogenic phenotype, and chondrogenic differentiation of MSCs in tissue engineering of cartilage [177]. Comparing normoxic to hypoxic conditions is of interest to determine the best culture conditions for the cells [59,178,179]. This parameter is yet to be assessed in EBB processes. 

Supplementation of the culture media with growth factors can also impact cell differentiation. Members of the TGF-β superfamily are essential regulators of chondrogenic differentiation during embryonic development in chondrogenesis and osteogenesis. The TGF-β superfamily is composed of 5 members (TGF-β1 to TGF-β5). TGF-β1 remains the most widely used and is known to stimulate the synthetic activity of chondrocytes and induce the chondrogenic differentiation of bone-marrow MSC, by decreasing the expression of type I collagen and increasing the production of type II collagen and aggrecan [180]. Bone morphogenetic proteins (BMPs) are glycoproteins of the TGF-β superfamily. BMP-2 is the most widely used in vitro to induce cartilage-type ECM production, with the synthesis of PGs and type II collagen [181]. Other factors such as IGF-1 showed an interest to maintain chondrocyte anabolic activities [182]. 

In extrusion-based constructs, culture media supplementation with growth factors is essential for the redifferentiation of embedded chondrocytes or MSC differentiation induction. TGF-β1 is used in many different studies involving embedded ACPCs [161] or MSCs [163,175]. Extruded gels can also be cultured in medium supplemented with TGF-β3 to induce chondrocyte redifferentiation [157] or MSC chondrogenesis [143,165], or in coculture substitutes containing both cell types [173]. For cartilage TE, BMP2 is usually associated with a TGF-β factor. A study aiming to compare TGF-β1, TGF-β3, BMP-2, or the association of these factors demonstrated the benefit of BMP-2 and TGF-β1 [60]. TGF-β2 was also used in 3D printed substitutes fed with chondrocytes, to support cartilaginous matrix formation [120].

### 5.7. Biomechanical Properties

Healthy articular cartilage needs to have biomechanical properties for physiological load bearing. The compressive modulus of native cartilage ranges from 240 to 1000 kPa but many hydrogels fail to meet this criterion [183]. Thus, 3D-printed cartilaginous biomechanical properties need to be investigated. Only a third of the studies presented in this review evaluated the compressive strength of the constructs either directly after printing [154], after some maturation time [173], or by comparing different time points [143,163,172]. Most commonly, the mechanical process used for evaluation of the properties are a uniaxial compressive test [154,155], unconfined compression test [143,158,162,172], dynamical mechanical compression test [163,173], or indentation [142]. The aim of those measures being to compare the tissue-engineered construct to native cartilage but also to evaluate the strength of different bioprinted biomaterials [162], to compare acellular constructs to cellularized ones [172], or even to evaluate the most efficient culture conditions for chondrogenesis [143]. The different biomechanical parameters such as compressive strength but also stiffness and elasticity need to be further investigated to better mimic native cartilage properties.

We resume all parameters discussed above, such as cell origins, densities, and viability, and chondrogenic and biomechanical properties in Table 2. 

## 6. Conclusions and Future Directions

Currently, extrusion-based 3D printing can be used to produce cartilaginous constructs. Future applications still present challenges and limitations. Most studies aim to produce standardized structures, generally cubes or rings, but in order to treat cartilaginous defects, the 3D constructs need to be adapted to the depth and shape of a unique lesion [93]. Furthermore, the biomaterial used to produce those structures needs to integrate with the native cartilage edges to form a strong and stable joint surface [142]. New noninvasive methods are being developed to assess cartilage thickness, composition, and functional integrity [184]. The most challenging requirement of TE is that the neosynthesized matrix must have sufficient stability to bear the physiological loads of the joint. Thus, numerous biomechanical parameters need to be assessed after 3D printing (compression modulus, deformation) [120,143,163]. The gelation process usually necessary to maintain printed construct integrity remains an obstacle to direct in situ printing. New methods were developed to directly crosslink the ink during the printing process [185] or to polymerize the bioink in situ [142]. The ideal cell type to use for cartilage TE is still controversial. The sole resident cells of cartilage present many disadvantages to in vitro expansion, such as in vitro dedifferentiation. Adult MSCs are a promising alternative. With good proliferation and differentiation potential, they offer new cell types and density possibilities for engineered constructs. Other cell types were extruded with subsequent evaluation of their chondrogenic properties, such as ATDC5 cells [186,187] or IPS cells [188]. Today’s research aims to create layered substitutes that reproduce the natural zonal organization of hyaline cartilage, by optimizing cell types and densities, biomaterials, and environmental factors (growth factors, oximetry), to mimic both the structural and biomechanical properties of the natural material. However, many barriers still remain for the clinical translation of those research [189].

## Figures and Tables

**Table 1:** Summary of the advantages of the various biomaterials used to date in extrusion-based 3D printing depending on the embedded cell types for cartilage tissue engineering.

Biomaterial	Advantages	Cell Types	References
Alginate	Biocompatible Good printability Ionic gelation Low cost	Chondrocytes	[117,119,155,157,158]
		BM-MSCs	[60,162,166]
		Chondrocytes + BM-MSCs	[173]
Gelatin	Biocompatible Non immunogenic Thermic gelation Biodegradable	Chondrocytes	[120,154]
		ACPCs	[161]
		BM-MSCs	[60,163,165,166,175]
		ADSC	[142,143,168]
		Chondrocytes + BM-MSCs	[173]
Hyaluronic acid	Biocompatible Promote proliferation Bio printability Chondrogenic signalling Chemical crosslinking Anti-inflammatory	Chondrocytes	[118]
		BM-MSCs	[165]
		ADSC	[142,143,168]
		Chondrocytes + BM-MSCs	[173]
Collagen	ECM component Good printability Promote cell adhesion Chondro-induction	Chondrocytes	[155,156]
Chondroitin sulphate	Component of ECM Anti-inflammatory Gelation by chemical modification	Chondrocytes	[118]
		BM-MSCs	[165]
		Chondrocytes + BM-MSCs	[173]
Nanocellulose	Biocompatibility Shear thinning properties High stiffness	Chondrocytes	[117,119,158]
		Chondrocytes + BM-MSCs	[171,172]
Agarose	Biocompatible High stability Thermic gelation Low cost	Chondrocytes	[155]
Fibrinogen	Biocompatible Strong 3D network Chemical crosslinking	BM-MSCs	[60]
dECM	Biocompatible Native ECM structure Biological cues Promote cell growth and differentiation	Chondrocytes	[108]
		BM-MSCs	[106]

NB: We presented only the studies using the 3D bioextrusion printing method, where the hydrogel-based bioink is directly seeded by the cells before printing. Therefore, we eliminated acellular printing methods, publications associating a PCL scaffold with hydrogels, and studies using extrusion coupled with post-printing molding.

**Table 2:** Comparison of cell viability and chondrogenic evaluation depending on cell types, origins, and densities used to date in extrusion-based processes for cartilage tissue engineering.

Cell Type	Bioink Components	Species	Cellular Density (cells/mL)	Cell Viability	Chondrogenic Evaluation				Reference
					Biochemical Assays	Gene Expression	Matrix Synthesis	Biomechanical Testing	
**Chondrocytes**	Silk fibroin Gelatin	Porcine	1 M	Good Viability	DNA and GAG content	*Col2*,* Sox9*,* ACAN* and *Col10*	H&E staining	Uniaxial compressive test	[154]
	Alginate Nanocellulose	Human	2 M	71.6–97.3%	N.D	N.D	N.D	Unconfined compression test	[158]
	Type II collagen	Rabbit	5 M 10 M 20 M	93%	DNA and GAG content	*Col1A1*,* Col2A1* and *ACAN*	H&E and Alcian blue staining Type I and II collagen and PRG4 IHC	N.D	[156]
	Alginate Nanocellulose	Calves	6 M	>65%	N.D	N.D	Type I and II collagen and Proteoglycan-HA IHC	N.D	[117]
	Hyaluronic acid Chondroitin sulphate	Bovine	6 M	91%	N.D	N.D	N.D	N.D	[118]
	Alginate Methylcellulose	Human	6.5 - 7 M	45–75%	GAG and type II collagen content	*Col2*,* ACAN*,* COMP*,* Col10*,* Col1* and *Sox9*	Aggrecan IF	N.D	[157]
	Sodium Alginate Agarose Type I collagen	Rat	10 M	70–95%	DNA and GAG content	*ACAN*,* Sox9*,* Col2A1* and *Col1A1*	H&E staining	Uniaxial compressive test	[155]
	GelMA	Equine	10-20 M	N.D	DNA and GAG content	N.D	Safranin-O/Fast green staining Type II collagen IHC	N.D	[120]
	Alginate Nanocellulose	Human	15 M	72.8–93%	N.D	N.D	N.D	N.D	[119]
	dECM	Rabbit	20 M	90–98%	GAG and collagen content	N.D	H&E, Safranin-O/Fast-green and Alcian blue staining	N.D	[108]
**ACPCs**	GelMA	Equine	20 M	>70%	DNA and GAG content	*Col1A1*,* Col2A1* and *PRG4*	Safranin-O staining Type I and II collagen IHC	N.D	[161]
**BM-MSCs**	Alginate, Gelatin and Fibrinogen	Human	1 M	>90%	N.D	*Col2A1*,* Col10A1*,* ACAN*,* VCAN*,* Sox9*,* COMP*,* ALP*,* BGLAP* and *OSX*	HES, Alcian blue, Alizarin red and Sirius Red staining Type II collagen IHC	N.D	[60]
	Hyaluronic acid	Human	3 M	Good viability	DNA and GAG content	*ACAN*,* Col2A1*,* Sox9*,* Col1A1*,* Col10A1* and *RunX2*	Safranin-O/Fast green staining Type I collagen IF	N.D	[103]
	GelMA	Human	4 M	Good viability	N.D	N.D	H&E, Alcian blue and picrosirius staining Type I and II collagen IHC	N.D	[175]
	Sodium alginate	Human	6 M	73–87%	N.D	N.D	Alizarin red, Sirius red and Safranin-O staining Type I and II collagen IHC	Unconfined compression test	[162]
	GelMA and alginate	Human	10 M	>80-85%	N.D	*Col1*,* Col2*,* Col10A1*,* ACAN*,* ALPL* and *BGLAP*	Type I, II and X collagen and aggrecan ICC	N.D	[166]
	dECm, silk-fibroin and PEG	Rabbit	10 M	>80%	DNA, GAG and collagen	*Col1*,* Col2*,* ACAN* and *Sox9*	Safranin-O and Masson’s trichrome staining	N.D	[106]
	Alginate, HAMA and CS-AEMA	Human	10–15 M	85–90%	N.D	*ACAN*,* Col1*,* Col2* and *Col10*	Type I, II and X collagen and aggrecan ICC	N.D	[165]
	HAMA and GelMA	Rat	20 M	>90%	DNA and GAG content	*Col2*,* Col1*,* Col10*,* Sox9* and *ACAN*	Alcian blue, H&E, Safranin-O staining	Dynamic mechanical compressive test	[163]
**ADSCs**	HAMA-GelMA	Human	2 M	97%	N.D	N.D	N.D	N.D	[168]
	HAMA-GelMA	Sheep	2.5 M	97%	N.D	N.D	Safranin-O/Fast green staining Type I and II collagen IHC	Indentation test	[142]
	HAMA-GelMA	Human	10 M	N.D	N.D	*Col2A1*,* Col1A2*,* ACAN*,* Sox9*,* RunX2* and *Col10A1*	Alizarin red, Safranin-O staining Type I, II and X collagen and proteoglycan IF	Unconfined compression test	[143]
**BM-MSCs + Chondrocytes**	NFC	Human	10 M	N.D	N.D	N.D	Alcian blue, Von Gieson and Safranin-O staining Type II collagen IHC	N.D	[171]
	NFC and alginate	Human	10 M	N.D	N.D	N.D	Alcian blue and Von Gieson staining Type II collagen IHC	Unconfined compression test	[172]
	GelMA, HAMA, CS-AEMA	Human	10 M	88–90%	N.D	*Col2A1*,* ACAN*,* Col1A1*,* Col10A1* and *ALPL*	Type I, II and X collagen and aggrecan IHC	Dynamic mechanical compressive test	[173]

N.D = No Data; Good Viability means that no precise value is available; *ACAN*: Aggrecan (gene), *BGLAP*: Osteocalcin (gene); *COMP*: Cartilage oligomeric matrix protein (gene); CS-AEMA: Chondroitin sulphate 2-aminoethyl Methacrylate; *Col1A1*: Collagen Type I Alpha 1 Chain (gene); *Col2A1*: Collagen Type II Alpha 1 Chain (gene); *Col1*: Collagen Type I (gene); *Col2*: Collagen Type II (gene), *Col10*: Collagen type X (gene); dECM: decellularized ExtraCellular Matrix; DNA: Deoxyribonucleic acid; GAG: Glycosaminoglycan; GelMA: Gelatin methacrylamide; H&E: Hematoxylin Eosin staining; HA: Hyaluronic Acid; HAMA: Methacrylated hyaluronic Acid; HES: Hematoxylin Eosin and Saffron staining; ICC: Immunocytochemistry, IF: Immunofluorescence; IHC: Immunohistochemistry; M: 10^6^ (for Million); NFC: Nanofibrillated Cellulose; PEG: Poly(Ethylene)-Glycol; *PRG4*: Proteoglycan 4 (gene); *RunX2*: Runt-related transcription factor 2 (gene); *Sox9*: Sex-determining region-related HMG-box9 (gene); and *VCAN*: Versican (gene). NB: We presented only the studies using the 3D bioextrusion printing method, where the hydrogel-based bioink is directly seeded by the cells before printing. Therefore, we eliminated acellular printing methods, publications associating a PCL scaffold with hydrogels, and studies using extrusion coupled with post-printing molding.

## Abbreviations

3D	Three Dimension
ACI	Autologous chondrocyte implantation
ACPC	Articular Chondroprogenitor Cells
ADSC	Adipose Derived Stem Cells
BM-MSC	Bone Marrow-derived Mesenchymal Stem Cells
BMP	Bone Morphogenetic Protein
CAD	Computer-Aided Design
CNTs	Carbon nanotubes
CS	Chondroitin Sulfate
dECM	decellularized ExraCellular Matrix
EBB	Extrusion-Based Bioprinting
ECM	ExtraCellular Matrix
GelMA	Gelatin Methacryloyl
HA	Hyaluronic Acid
IGF	Insulin-like Growth Factor
IPSc	Induced Pluripotent Stem cells
MSC	Mesenchymal Stem Cells
OA	OsteoArthritis
PCL	Polycaprolactone
PEG	Poly(Ethylene)-Glycol
PG	Proteoglycan
PGA	Polyglycolic acid
TE	Tissue engineering
TGF-β	Transforming Growth Factor Beta
UV	UltraViolet
